# The Medical Profession of the United Kingdom

**Published:** 1888-12

**Authors:** 


*The Medical Profession of the United Kingdom.* The Carmichael Prize Essays. The first Prize Essay by WALTER RIVINGTON, M.B., F.R.C.S., London; the second by THOMAS LAFFAN, M.C.P.I., Cashel.

Although Mr. Rivington undoubtedly deserved the first prize awarded by the Council of the Irish Royal College of Surgeons, on account of the wider grasp and greater fulness of his essay, that of Dr. Laffan is, considering his opportunities as a remote country practitioner, very remarkable for the power and force of his language and the closeness and justice of his criticism.

We should like to see each of these books in the hands of every member of our profession. The ponderous volume of Mr. Rivington, containing 1200 octavo pages, may profitably be compared with the more handy volume of Mr. Laffan, containing only 385 pages; the two give full and accurate views of ourselves as we are in education, in practice, and in government. To the criticisms we would specially direct attention. The smug callousness of our General Medical Council; the complacent incapacity of the self-elected Committee of Council of the British Medical Association; the shifting instability and bewildering variety of medical education and examination; and a host of other matters are dealt with fairly and competently. We commend these volumes to the favourable notice of our readers.

